# Do people living in disadvantaged circumstances receive different mental health treatments than those from less disadvantaged backgrounds?

**DOI:** 10.1186/s12889-020-08820-4

**Published:** 2020-05-11

**Authors:** Clarissa Giebel, Rhiannon Corcoran, Mark Goodall, Niall Campbell, Mark Gabbay, Konstantinos Daras, Ben Barr, Tim Wilson, Cecil Kullu

**Affiliations:** 1 Institute of Population Health Sciences, University of Liverpool, Liverpool, UK; 2 NIHR ARC NWC, Liverpool, UK; 3 Cheshire and Wirral Partnership NHS Foundation Trust, Chester, UK; 4 Merseycare NHS Foundation Trust, Prescot, UK; 5 School of Environmental Sciences, University of Liverpool, Liverpool, UK

**Keywords:** Mental health, Health inequalities, Deprivation, Socio-economic status, Medication; therapy, Health care access

## Abstract

**Background:**

Socio-economic status (SES) has been linked to treatment outcomes for mental health problems, whilst little to no literature has explored the effects of SES on access to both medication and psychological therapy. The aim of this study was to explore whether access to mental health treatments differed by SES.

**Methods:**

The North West Coast Household Health Survey (HHS) collected data from residents aged 18+ from across 20 disadvantaged and 8 less disadvantaged neighbourhoods in 2015, and from 20 disadvantaged neighbourhoods in 2018. Logistic regression was used to explore the effects of SES on access to treatment (medication, psychological therapy) for people who had experienced mental health problems in the past 12 months.

**Results:**

Of 6860 participants, 2932 reported experiencing mental health problems in the past 12 months. People from more disadvantaged backgrounds experienced greater rates of anxiety and depression. Anti-depressant and anti-psychotic medication treatment was significantly more common in residents with lower SES, as well as counselling. Regression analysis showed that residents from more disadvantaged neighbourhoods who reported mental distress were more likely to receive medication.

**Conclusions:**

This appears to be the first study showing higher levels of treatment with medication and psychological therapy in people from disadvantaged backgrounds. Future research needs to address the underlying factors associated with increased mental health treatment uptake in people from lower socio-economic backgrounds.

## Background

Depression and anxiety are reported to affect 1 in 6 people in the UK [1]. Commonly co-morbid, both can be treated with psychotropic and/or psychological or talking therapies. However, a recent report by the World Health Organisation has highlighted that of those with a diagnosis of anxiety, mood, or substance disorders, only 14, 22, and 37% receive treatments in low-, middle-, and high-income countries, respectively [2].

Socio-economic status (SES) is found to be linked to differences in treatments in both general health care usage [3], and in terms of mental health - both for inpatient and outpatient services [4–7]. SES as measured by the Index of Multiple Deprivation (IMD) [7] comprises a number of factors, including education, ethnicity, gender, income, debt, and housing, representing a relative measure of deprivation by measuring of neighbourhood, not individual level, deprivation. Looking at access to medication as one form of mental health treatment, Halonen and colleagues [5] reported socio-economic inequalities in access to anti-depressants in Finland in that people living outside of the capital as well as those with poorer levels of education were more likely to access the older anti-depressants medications associated with more side effects. In contrast, those living in the capital and with higher levels of education were more likely to receive the newest types of anti-depressant medication. This supports previous evidence showing that people with lower SES are more likely to receive anti-depressants and to report higher levels of anxiety and depression [8]. It also corroborates a recent report by the World Health Organisation showing that higher levels of education are linked to higher levels of treatments across low-, middle-, and high-income countries [2]. However, it appears that to date no study has compared access to different types of mental health treatments in the form of medication and psychological therapy by SES.

With the aim of examining socio-economic variations in healthcare utilisation, a longitudinal public health survey was implemented across 28 neighbourhoods in the North West Coast (NWC) of England, one of the most disadvantaged regions in the country [7]. The NWC Household Health Survey (HHS) has collected information on a number of demographic and neighbourhood level characteristics, health, mental health, lifestyle, social capital, and health care utilisation. Previous analysis of the HHS has shown that being unemployed and living in poor quality housing, as well as living further from a GP practice, were linked to increased rates of Accident and Emergency (A&E) attendance [9]. This may suggest sub-optimal health management by not approaching primary care services first, but instead attending A&E. Furthermore, comorbid mental health problems have also been linked to increased healthcare utilisation in general [10]. This research has corroborated the existence of social inequalities in accessing general healthcare services [3], whilst not specifically investigating mental health treatment.

This study had two aims: (a) to explore the relationship between mental health (anxiety and depression) and SES; and (b) to ascertain whether people reporting depression and/or anxiety from more disadvantaged backgrounds receive different types of mental health treatments than those from less disadvantaged backgrounds. Based on previous evidence [11], we hypothesised that higher levels of deprivation were associated with higher levels of anxiety and depression. We hypothesised that people who live in areas with higher levels of deprivation were more likely to receive medication as opposed to psychological treatment, whereas people who live in less disadvantaged areas would be more likely to receive psychological treatments.

## Methods

### Participants and recruitment

The North West Coast Household Health Survey data have so far been collected in two waves from 20 disadvantaged neighbourhoods across the NWC region, with Wave 1 also containing data from eight less disadvantaged neighbourhoods (see Giebel C, McIntyre JC, Alfirevic A, et al. The longitudinal NIHR ARC North West Coast Household Health Survey: Exploring health inequalities in disadvantaged communities. BMC Public Health. submitted. for further details). Wave 1 and 2 were implemented in September to December 2015 and 2018, respectively. Sampled neighbourhoods were identified by local authorities based on IMD score and their in-depth knowledge of areas of deprivation. Data were collected by an independent agency (BMG), which knocked on the doors of households during day-time hours. Only one member per household took part in the survey, which lasted approximately 45 min. Participants had to be aged 18 or over to take part in the survey. Those Wave 1 participants who had given their consent to be approached again in Wave 2 were first approached by researchers. Where people had moved or were not at home, or no longer willing to participate, new households were approached to take part.

The HHS collects longitudinal data, yet not everyone who participated in Wave 1 also participated in Wave 2. In addition, new participants were also taking part in Wave 2. For this study, only data from participants who had participated at one time point, or at the first time point if they took part in both waves, were included. Thus, of the total 7731 cases, 871 were repeated, resulting in 6860 cases included in the overall analysis. Of those, 2932 participants had stated to have experienced a mental health issue in the past 12 months.

Ethical approval was obtained from the University of Liverpool (Ref: RETH000836). Participants provided written informed consent prior to taking part in the study.

### Public involvement

Three members of the public were involved in the design of the research question, interpretation of the analysis, and in the dissemination. They attended regular team meetings and provided feedback on drafts of this manuscript [12], in addition to writing a lay summary of the findings for the general public. Public advisers were reimbursed according to NIHR INVOLVE [13] guidelines for each activity and meeting, and had their travel expenses reimbursed.

### Data selection

The HHS includes a wide variety of information on demographics, healthcare utilisation, lifestyle, social capital, medical issues, and socio-economic and neighbourhood factors. A detailed overview of the type of collected data is submitted elsewhere. For the purpose of this analysis, we included the following variables: Demographic characteristics (age, gender, ethnicity (white, mixed/multiple ethnic groups, Asian/Asian British, Black/African/Caribbean/Black British, other), education (educational qualification (yes/no), level of highest qualification), income (various options for weekly, monthly, and/or annual income)), living situation (alone vs. with others), Index of Multiple Deprivation (IMD) quintile – with ‘5’ indicating the most disadvantaged neighbourhoods, and ‘1’ indicating the least disadvantaged neighbourhoods; the Generalised Anxiety Disorder Assessment 7 (GAD-7) [14], with scores ranging from ‘0’ to ‘21’, and a score of 10 and above identifying moderate anxiety [15], and the Personalised Health Questionnaire 9 (PHQ-9) [16] for depression, with scores ranging from ‘0’ to ‘27’ and a cut-off score of 10 used to identify moderate depression [15]. Regarding the IMD, data for the HHS were collected in 2015 and 2018, and IMD ratings were based on the 2015 weightings for each LSOA. Comparing more recent IMD (2019) quintile scores with those from 2015 as used in the survey, only three LSOAs changed from Quintile 4 to Quintile 5 from 2015 to 2019, therefore not having an effect on the overall analysis.

Participants were asked about whether they had experienced mental health problems in the past 12 months with a binary outcome provided (1 – yes; 0 – no). This therefore differs from the data obtained on participants’ levels of anxiety and depression, which are assessed via established clinical measures.

Types of mental health treatment received in the past 12 months was reported by the participant, in terms of medication (antidepressants or antipsychotics) and psychological / talking therapy (counsellor and psychological therapist).

### Data analysis

Demographic characteristics were analysed using frequency analysis. Binary logistic regression analysis of the whole sample was employed to explore the effects of IMD quintile, ethnicity, gender, age, and living situation (independent variables) on anxiety (as measured by the GAD-7) and on depression (as measured by the PHQ-9). Scoring above the cut-offs in the GAD-7 and PHQ-9 was used as the binary outcome variable (‘1’ - yes; ‘0’- no) such that groups comprised those reporting no or mild depression/anxiety and those reporting moderate or severe depression/anxiety.

On a sub-sample of participants who reported having experienced mental health issues in the past 12 months, binary logistic regression analysis was used to assess the effects of IMD quintile, anxiety, and depression on type of treatment as outcome variable (Model 1: Medication; Model 2: Psychological therapy). For this purpose, IMD quintiles 1–4, due to the low sample size, were merged into one factor, to compare the effects of IMD quintile 5 vs 1–4.

Significance value was set at *p* < .05, and data were analysed using SPSS 25.

## Results

### Sample characteristics

Table 1 shows the sample characteristics of the total and the sub-sample. Of the 6860 who participated in Wave 1 or 2, the majority of participants were female (58.7%), from a White ethnic background (94.7%), and lived with others (57.6%). Approximately 24% had a degree, with others stating to have another type of qualification. However, many participants failed to report their degree levels (53.7%). The majority of residents lived in some of the most disadvantaged neighbourhoods as measured by the IMD quintile (74.5%). Two thousand nine hundred thirty-two people (42.7%) reported having experienced mental health issues in the past 12 months.

**Table 1:** Demographic characteristics of residents with mental health problems and total sample

Demographics	Residents with mental health problems (***n*** = 2932)	Total sample (***n*** = 6860)
**N (%)**		
Age Group		
18–24	142 (4.8)	731 (10.7)
25–34	267 (9.1)	1284 (18.7)
35–44	362 (12.4)	1076 (15.7)
45–54	513 (17.5)	1051 (15.3)
55–64	570 (19.4)	989 (14.4)
65–74	566 (19.3)	957 (14.0)
75+	511 (17.4)	762 (11.1)
Gender		
Female	1720 (58.7)	3836 (55.9)
Male	1212 (41.3)	3024 (44.1)
Education^1^		
Degree level	319 (23.5)	1020 (26.5)
Another qualification	1039 (76.5)	2822 (73.5)
Ethnicity		
White	2768 (94.7)	6116 (89.5)
Mixed/Minority ethnic background	158 (5.3)	744 (10.5)
Living situation		
Living alone	1243 (42.4)	2204 (32.1)
IMD Quintile		
1	130 (4.4)	360 (5.2)
2	72 (2.5)	250 (3.6)
3	150 (5.1)	384 (5.6)
4	397 (13.5)	1074 (15.7)
5	2183 (74.5)	4792 (69.9)
Mental health problems^2^		
Depression	827 (28.2)	1165 (17.0)
Anxiety	665 (22.7)	890 (13.0)
Mental health medication	742 (27.7)	856 (17.7)
Antidepressant usage	721 (26.9)	832 (17.2)
Antipsychotic usage	78 (2.9)	83 (1.7)
Psychological therapy	276 (9.5)	349 (5.1)
**Mean (SD)**		
Age	56 (18)	48 (19)
PHQ-9 score	7.0 (6.8) [0–27]	4.7 (5.9) [0–27]
GAD-7 score	5.5 (6.1) [0–21]	3.6 (5.1) [0–21]

**Legend***GAD-7* Generalised Anxiety Disorder Assessment 7; *IMD* Index of Multiple Deprivation; *PHQ-9* Personalised Health Questionnaire 9

^1^Many participants did not provide an answer to this question (53.7%)

^2^Mental health problems were defined as when scores on the PHQ-9 or GAD-7 were 10 or higher

**Fig. 1:**
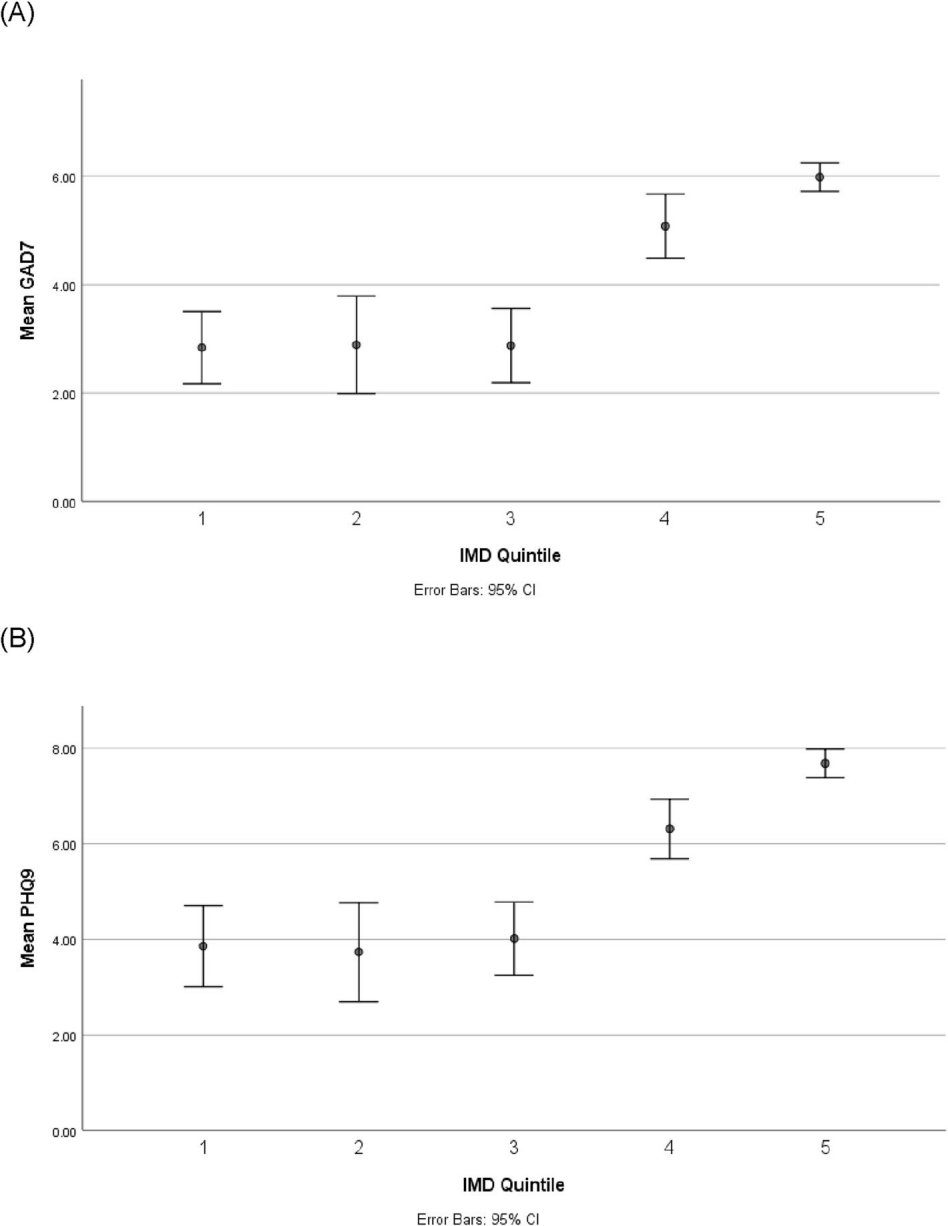
Anxiety and depression by socio-economic status. Based on sub-sample who have experienced mental health problems in the past 12 months. Quintile 1 (least disadvantaged) to Quintile 5 (most disadvantaged)

Based on the PHQ-9 and GAD-7 cut-offs for moderate depression and anxiety, 17.0% of the sample experienced depression, and 13.0% experienced anxiety. This was higher in those who reported having experienced mental health problems in the past 12 months [Depression: 28.2%; Anxiety: 22.7%]. Nine hundred fifty-seven participants who had experienced mental health problems in the past 12 months scored above the cut off on the GAD-7 and/or PHQ-9 for anxiety and depression, respectively, representing 32.6% of all those who had experienced mental health problems in the past 12 months. Figure 1 shows the proportion of people with anxiety and depression of this sub-sample.

### Factors associated with depression and anxiety in the whole sample

Binary logistic regression analysis showed that IMD Quintile (*p* = .000–.036) (except Quintile 4 (*p* > .05)), living situation (*p* < .05), and age (*p* < .001) were significant determinants of experiencing depression. Residents from less disadvantaged neighbourhoods, those living with others, and increased age were less likely to have depression. Gender and education were not significant in this model (*p* < .05).

In a separate regression model, IMD Quintile 1 and 2 (p_Quintile1_ < .001; p_Quintile2”_ < .05), age (*p* < .001), and living situation (*p* < .05) were also found to be significant determinants of anxiety. Those living in less disadvantaged neighbourhoods, living with others, and increased age were less likely to have anxiety. Gender and education were not significant in this model (*p* < .05).

Table 2 shows the outcomes of the regression analyses.

**Table 2:** Logistic regression models with depression and anxiety as outcome variables for the whole sample

Independent variable	B	S.E.	***p***-value	Exp (B)	95% Confidence Intervals for Exp(B)	
**Depression**						
Constant	1.239	.269	**.000**	3.453		
IMD Quintile 1	−1.306	.386	**.001**	.271	.127	.577
IMD Quintile 2	−.888	.424	**.036**	.412	.179	.945
IMD Quintile 3	−1.160	.389	**.003**	.313	.146	.672
IMD Quintile 4	−.160	.181	.378	.852	.598	1.216
Age	−.031	.004	**.000**	.970	.962	.978
Gender	−.189	.132	.154	.828	.639	1.073
Living alone	−.409	.146	**.005**	.664	.499	.884
Education	−.275	.160	.084	.759	.555	1.308
**Anxiety**						
Constant	.972	.286	**.001**	2.527		
IMD Quintile 1	−1.988	.598	**.001**	.137	.042	.442
IMD Quintile 2	−1.919	.732	**.009**	.147	.035	.616
IMD Quintile 3	−.655	.374	.080	.519	.250	1.080
IMD Quintile 4	.062	.188	.740	1.064	.736	1.538
Age	−.033	.005	**.000**	.968	.959	.977
Gender	−.151	.143	.289	.860	.650	1.137
Living alone	−.339	.157	**.030**	.712	.524	.968
Education	−.341	.175	.051	.711	.505	1.001

**Note:** IMD Quintiles compared to Quintile 5 (most disadvantaged), *p*-values in bold indicate significance

### Medication usage by socio-economic background

**Fig. 2:**
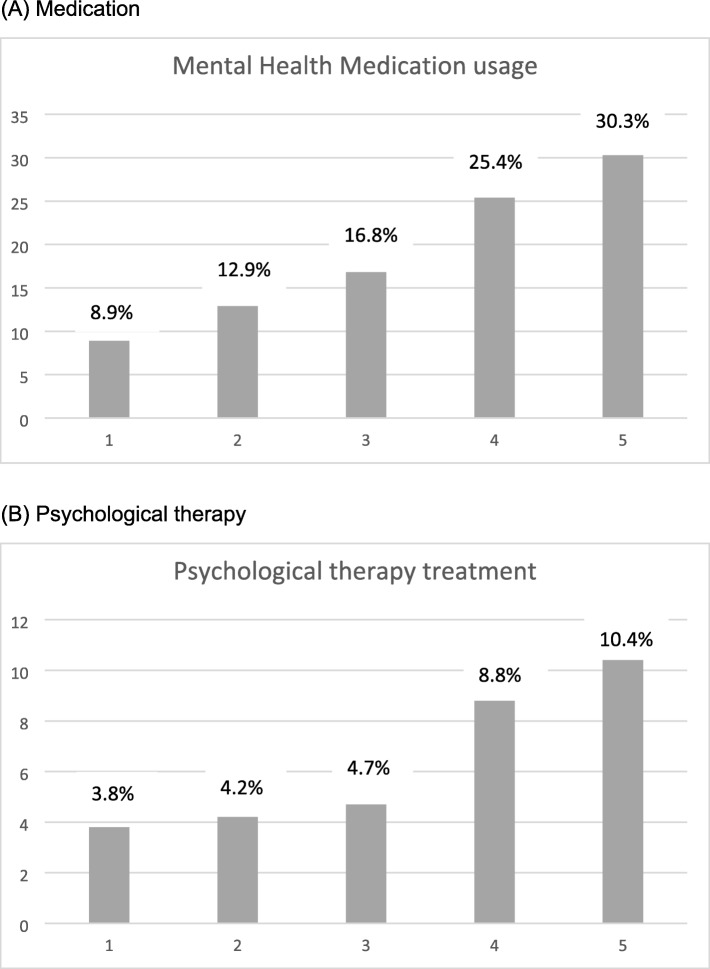
Mental health treatments by IMD Quintile. Based on sub-sample who have experienced mental health problems in the past 12 months. Percentage of (**a**) medication and (**b**) psychological therapy within each IMD quintile

For those who have experienced mental health problems in the past 12 months (*n* = 2932), 742 (27.7%) were using psychotropic medication, with antidepressants being more frequently used (26.9%) than antipsychotics (2.9%). Figure 2 shows the proportion of people who were using mental health medication by IMD quintile. Over 30% of people from the most disadvantaged quintile (5) were using medication for their mental health problems, as opposed to only 8.9% and 12.9% in those living in quintiles 1 and 2, respectively. In terms of polypharmacy and non-mental health medication, 41.4% (*n* = 1469) were using five or more different medications (Median 4). Average anti-depressant usage within the 20 disadvantaged neighbourhoods was 1.5 items per resident, and average national anti-depressant usage was 1.12 items per person.

One hundred eighty participants (6.1%) received both medication and psychological therapy.

### Determinants of mental health treatments in those with mental health problems

Figure 2 shows mental health treatment by IMD quintile. Chi^2^- tests showed that receiving psychological therapy [x^2^(42913) = 13.641, *p* < .01] and receiving medication [x^2^(42681) = 42.436, *p* < .001] varied significantly by IMD quintile. Receipt of psychological therapy (10.4%) and use of medication (30.3%) was highest in those living in the most disadvantaged neighbourhoods (Quintile 5).

Binary logistic regression models showed that IMD quintile was a significant determinant of accessing mental health medication, but not psychological therapy. Participants from the most disadvantaged neighbourhoods (Quintile 5) were significantly more likely to access mental health medication (*p* = .002). For both mental health medication and psychological therapy, usage was significantly higher for those who had anxiety (*p* < .001; *p* < .001) and depression (*p* < .001; *p* < .001), as measured with the GAD-7 and the PHQ-9. Table 3 shows details of the logistic regression models.

**Table 3:** Logistic regression models with mental health treatments as outcome variables

Independent variable	B	S.E.	***p***-value	Exp (B)	95% Confidence Intervals for Exp(B)	
**Mental health medication**						
Constant	−1.889	.109	**.000**	.151		
IMD Quintile 5 vs 1–4	.357	.117	**.002**	1.429	1.135–1.798	
Anxiety	1.119	.124	**.000**	3.063	2.404–3.903	
Depression	.981	.118	**.000**	2.667	2.118–3.359	
**Psychological therapy**						
Constant	−3.124	.160	**.000**	.044		
IMD Quintile 5 vs 1–4	.211	.169	.212	1.234	.887–1.718	
Anxiety	.981	.170	**.000**	2.668	1.912–3.722	
Depression	.872	.171	**.000**	2.392	1.710–3.347	

**Note:** IMD Quintiles compared to Quintile 5 (most disadvantaged), *p*-values in bold indicate significance

## Discussion

This is one of the first studies showing that IMD is linked to variations in receipt of mental health treatment, including both medication and psychological therapy, separately. Specifically, people from more disadvantaged backgrounds were more likely to receive either medication or psychological therapy as forms of treatments compared to people from less disadvantaged backgrounds, thereby only partly confirming our initial hypotheses.

Lower SES has been established to be linked to lower levels of mental well-being [11, 17]. However, the limited previous research has only explored the effects of SES on either the utilisation or outcomes of one form of treatment – either medication [18] or psychological therapy [19–21]. In a large British Household survey, Jokela and colleagues [21] found that higher SES was linked to being less likely to have mental health issues and to access public psychotherapy services, but instead more likely to access private services. Whilst they did not investigate access to anti-depressants and anti-psychotics by SES, Jokela et al. [21] furthermore showed that the use of publicly provided psychotherapy services has improved over an 18-year period in those from low socio-economic backgrounds. Findings from the present study slightly go against previous results, as in the present study, people from lower socio-economic backgrounds were not significantly more likely to access psychological therapy based on findings from regression analysis, and when accounting for anxiety and depression. However, people from disadvantaged backgrounds were more likely to access medication. When not controlling for anxiety and depression though, both models indicated that people living in more disadvantaged neighbourhoods were significantly more likely to receive access to either form of treatment. With the NWC HHS not distinguishing between publicly and privately provided psychological therapy, it is not possible to say however which type of psychological treatments people in each neighbourhood were accessing. It is possible that services such as Improving Access to Psychological Therapy (IAPT) [22] were utilised by participants, as opposed to privately accessed services.

IAPT was set up 10 years ago to meet the psychological needs of the population, with the aim of providing recovery of mental health issues in 50% of those treated [22], which was found to be effective [23]. A recent evaluation of 144 IAPT services across England has shown that whilst people from lower socio-economic backgrounds have a higher prevalence of mental health issues, they are less likely to access IAPT, and thus publicly-funded psychological therapy services, compared to those from less disadvantaged backgrounds [19]. However, as Delgadillo et al. [19] categorised the most disadvantaged quintile as IMD Quintile ‘1’ and the least disadvantaged as Quintile ‘5’, the authors did not adhere to the official guidance on IMD quintiles. This is because Quintile 1 is the most disadvantaged quintile, and Quintile 5 the least disadvantaged quintile. Findings on the prevalence of mental health issues are in line with expected values though, and so we can assume that the reported higher access gap for psychological therapy was correct. The findings reported here contrast with Delgadillo et al’.s [19] study by showing higher levels of access to psychological therapy in those from the most disadvantaged neighbourhoods. It is likely that these variations were due to the fact that Delgadillo et al. [19] specifically investigated access to IAPT services, whereas the present study explored any form of psychological/ talking therapy. However, Delgadillo and colleagues [19] focused specifically on people that were referred for access, as opposed to the present study which explored people using or not using either form of mental health treatment, so that a comparison needs to be considered with caution.

Overall, access to mental health care in the UK has been found to have improved, both for anti-depressant medication and for psychological therapy [23]. This is corroborated by the findings reported here for the NWC, but only for those from lower socio-economic backgrounds. However, compared to the national average, residents from the 20 disadvantaged neighbourhoods in the survey looked as though they could be receiving higher prescription rates of anti-depressant medication. According to national guidance, people in the milder stages of depression should be referred to psychological therapy rather than medication, depending on individual circumstances [24]. However, in moderate to severe depression, patients are recommended to receive a combination of both. Importantly, the HHS did not capture what was offered in terms of mental health treatments. Thus, it is not possible to state whether people were prescribed anti-depressants (or anti-psychotics) instead of being offered psychological therapy.

Taking a more international view, the WHO has shown that higher levels of education for example are linked to better treatment in low-, middle-, and high-income countries [2], whilst disparities in access to mental health treatments are also widely reported in the US for example [25]. This is corroborated by findings from Lauzier and colleagues [26], where people living in more disadvantaged neighbourhood were slightly more likely to receive access to antidepressant treatment than those living in less disadvantaged neighbourhoods in a region in Canada, whilst no variations between neighbourhood deprivation and quality of antidepressant treatment were found for those publicly insured. Future research needs to explore variations in treatment options and uptake, based on socio-economic background, not only in the UK, but internationally, taking into consideration different insurance backgrounds for each country, and whether these might also facilitate more equal access.

### Limitations

This study is subject to some limitations. The NWC HHS collected self-reported data on experiences of mental health issues in the past 12 months at two different time points, not clinical diagnoses. However, the GAD-7 and the PHQ-9 are used in the clinical assessment of anxiety and depression and in measuring clinical therapy outcomes [27, 28], and therefore are suitable measures to establish presence of anxiety and depression. Data were collected at two different time points 3 years apart, which may have been a limitation, as data were used both from Wave 1 and Wave 2, but only one data entry per participant. However, 3 years is a standard time window between different points of data collection in longitudinal studies, and no major changes to health policy were introduced which would have biased the data. Moreover, whilst the total sample constituted over 6800 and 2900 cases for both types of analyses, respectively, Quintile 5 was the most populated due to the focus of the HHS on people living in disadvantaged backgrounds. Thus, samples in Quintiles 1 to 4 were much smaller and comparing the effects of treatment access by quintile may have been confounded by unequal sample sizes. Thus, the sample and the findings are also not representative of the general population, due to the greater representation of people from more disadvantaged backgrounds. Although SES was found to be a significant determinant of receipt of mental health treatments, due to the nature of this cross-sectional cohort study however, it is not possible to imply causation. Lastly, whilst education was not included in the main analysis, it is to be noted that nearly 54% of participants had missing data on this variable. This is likely to be the result of participants from these more disadvantaged backgrounds potentially having lower levels of education and potentially not feeling comfortable sharing this information. This may have slightly biased the results. However, as the main focus was set on neighbourhood deprivation, these missing data were not as relevant as IMD quintile data, which was complete. Future research needs to explore the underlying reasons of these variations in mental health treatments by SES.

## Conclusions

This study provides some of the first insights into the relationship between SES and access to mental health treatments, showing how people from the most disadvantaged backgrounds are more likely to access both medication as treatment. Whilst this study provides important evidence in this burgeoning field of work, further research needs to explore the underlying causes of this increase in access and utilisation of both types of mental health treatments in a larger sample, by ensuring equal samples of both those living in disadvantaged and in less disadvantaged neighbourhoods.

## Data Availability

Users can obtain access to the ARC NWC HHS data files after submitting a brief proposal (including agreement to HHS’ conditions of use) at [info@pldr.org]. Users will also be required to outline which version of the survey dataset they wish to access, data security arrangements in place and how they meet the criteria for access. Access to the data will be authorized following approval from the PLDR governance board.

## Abbreviations

GAD-7: Generalized Anxiety Disorder 7
HHS: Household Health Survey
IMD: Index of Multiple Deprivation
MANOVA: Multivariate Analysis of Variance
NWC: North West Coast
PHQ-9: Personal Health Questionnaire 9
SES: Socio-economic status
